# Upper Gastrointestinal Bleeding as the First Presentation of Eosinophilic Gastrointestinal Disease

**DOI:** 10.1097/PG9.0000000000000017

**Published:** 2020-11-11

**Authors:** Elif Ozdogan, Latife Doganay Caglayan, Ozlem Mizikoglu, Cigdem Arikan

**Affiliations:** From the * Department of Pediatric Gastroenterology, Hepatology and Nutrition, Koc University School of Medicine, Istanbul, Turkey; † Department of Pathology, Kent Hospital, Izmir, Turkey; ‡ Organ Transplantation Center, Liver Transplantation, Koc University School of Medicine, Istanbul, Turkey; § Research Center for Translational Medicine (KUTTAM), Koc University, Istanbul, Turkey.

**Keywords:** upper GI bleeding, children, eosinophilic esophagitis, budesonide, hematemesis, esophageal ulcer

## Abstract

Gastrointestinal bleeding is a rare but potentially life-threatening manifestation of eosinophilic gastrointestinal disorders (EGIDs). Here, we describe a case series comprising 5 pediatric patients between 7 and 12 years of age, who presented to the emergency department with hematemesis and were subsequently diagnosed with EGID. Accompanying allergic history, peripheral eosinophilia, and total IgE elevation were common. Despite a more severe presentation, response to medical and dietary therapy was favorable. A comprehensive review of the literature revealed 26 other cases with similar findings that reinforced the importance of prompt recognition and early dietary and immunomodulating therapy in the control of this disease.

**What Is Known**

- Eosinophilic gastrointestinal disorders (EGID) are characterized by the eosinophilic inflammation and associated dysfunction of the gastrointestinal (GI) tract.
- Symptoms of EGID are nonspecific and may include feeding difficulties, failure to thrive, nausea, vomiting, abdominal pain, and diarrhea.

**What Is New**

- Upper GI bleeding is not an uncommon initial presentation of EGID, but the data about the characteristics and prognosis of these patients are scarce.
- Initial treatment with intravenous proton pump inhibitors and systemic steroids allows efficient control of bleeding.
- Long-term clinical and histologic response to dietary and medical therapy is favorable in EGID-associated GI bleeding.

**E**osinophilic gastrointestinal diseases (EGID) are a heterogeneous group of disorders that are characterized by localized eosinophilic inflammation involving different parts of the gastrointestinal (GI) tract and subsequent GI dysfunction, in the absence of other known causes of tissue eosinophilia ^(1)^. They are considered rare diseases with prevalence rates ranging between 10–57/100.000 for eosinophilic esophagitis (EoE) and 3.3–8.4/100.000 for nonesophageal EGIDs. However, epidemiological studies have shown that the incidence rates of both EoE and Eosinophilic gastritis/Eosinophilic gastroenteritis (EG/EGE) have been steadily increasing at a global level (2).

Clinical presentation varies widely in accordance with age as well as site and depth of eosinophilic infiltration. Nevertheless, current guidelines advise an open-minded when EGIDs are suspected due to the nonspecificity and high variability of symptoms (1). Accordingly, EGIDs can rarely present with upper (UGIB) and lower (LGIB) gastrointestinal bleeding in severe cases. However, the literature on this presentation is currently limited to case reports (3–5).

Here, we report a case series comprising 5 pediatric patients diagnosed with EoE or EG/EGE following a presentation with hematemesis, with an emphasis on clinical and laboratory features suggestive of EGID, diagnostic challenges, and treatment outcomes. To our knowledge, the current report represents the first-case series describing EGID-associated GI bleeding.

## MATERIALS AND METHODS

We performed a retrospective review of the medical charts of 5 pediatric patients with EGID, whose initial presentation was hematemesis. Demographic, clinical, endoscopic, and histologic data of the patients were collected from medical records and charts. The diagnoses of EoE and EG/EGE were made according to the following peak tissue eosinophil counts: ≥15 eosinophils/high power field (hpf) in the esophagus, ≥30 eosinophils/hpf in the stomach, and ≥50 eosinophils/hpf in the duodenum (6, 7). Informed consent was obtained from all patients, and the study was approved by the Institutional Review Board (Ethics Committee) at Koc University.

## RESULTS

### Cases With EoE

Three patients (cases 1–3) between 8 and 12 years of age presented to the emergency department with severe hematemesis (Table 1). Before their presentation, they were suffering from recurrent abdominal pain (AP). All were previously treated with antiacid treatment without any improvement. Atopic history included concurrent allergic rhinitis in 3 and allergic asthma in 1. Laboratory findings were significant for anemia (1), elevated total IgE levels (3), and peripheral eosinophilia (PE) (2). Upper GI endoscopy revealed esophageal linear furrowing with white plaques (3), exudative hemorrhagic lesions (3), and esophageal ulcers (1). Histologic examination of the biopsy specimens showed eosinophil infiltration ≥15 eosinophils/high power field and eosinophilic abscesses.

**TABLE 1.** Clinical, demographic, laboratory, endoscopic, and histologic characteristics of the patients

Case	1	2	3	4	5
Age	12	10	8	14	7
Gender	F	M	M	F	M
Presentation	Hematemesis, abdominal pain	Hematemesis, severe vomiting	Hematemesis, abdominal pain	Hematemesis, abdominal pain	Hematemesis, abdominal pain
Symptom duration (d)	1	3	4	2	3
HPI	Recurrent abdominal pain	Recurrent abdominal pain	Recurrent abdominal pain and feeding refusal for 6 mo	Constipation treated with PEG	Recurrent abdominal pain for 1 yr
Atopy	AR	AR, asthma	AR	AD (resolution at age 4)	AR, AD (between ages 2 and 4)
WBC (/mm^3^)	7340	6400	8200	14,000	11,000
Hb (g/dL)	11.5	9	12.1	9.2	7
Platelet (/mm^3^)	326,000	346,000	328,000	468,000	265,000
PE (%)	2	10	5.8	4	12
CRP (mg/L)	2	3	Negative	24	18
Total IgE (IU/ml)	40	430	164	342	210
SPT	Rice, oat, sesame, fish, tomato	Milk, meat, pistachio	House dust mite, nut, egg	Molds, pollens	Negative at diagnosis and at 4 yrs
Ultrasonography	N/A	N/A	N/A	Normal	Normal
Endoscopy	Linear furrowing with white plaques; exudative hemorrhagic lesions	Linear furrowing with white plaques; esophageal ulcers	Linear furrowing with white plaques; exudative hemorrhagic lesions	Mucosal edema and severe hyperemia in greater curvature of stomach; large sheet erosion in antrum	Mucosal edema and severe hyperemia in greater curvature of stomach; ulcer
Histology	EoE	EoE	EoE	EG (98 eo/hpf)	EG (126 eo/hpf)
Therapy	PPI; steroid IV 1 mg/kg/d for 3 d; OVB for 6 mo; ED for 3 mo	PPI; steroid IV 1 mg/kg/d for 3 d; OVB for 12 mo; ED for 3 mo	PPI; steroid IV 1 mg/kg/d for 3 d; OVB 6 mo; ED for 2 mo	PPI; steroid IV 1 mg/kg/d for 3 d; crushed budesonide; ED milk-wheat for 3 mo	PPI; steroid IV 1 mg/kg/d for 3 d; crushed budesonide; ED milk and egg for 2 mo
Outcome	4 yr of follow-up No recurrence of bleeding At 2 yr of follow-up patient underwent EGD due to postnasal drip and sensation of globus. Esophageal biopsies revealed PEC of 10 eos/hpf with papillary elongation. He received OVB for 6 mos with ED (milk, rice, sesame, seafoods) for 3 mo. His symptoms disappeared within 1 mo and did not recur for 2 yr, after which he was lost to follow-up.	7 yr of follow-up No recurrence of bleeding presented with poor appetite and symptoms of reflux 3 yr after the initial diagnosis. EGD and biopsy revealed EoE. He received full-dose OVB for 6 mos and half-dose OVB for another 6 mos. He has been symptom-free for 3 yr after the second treatment.	2 yr of follow-up No recurrence of bleeding No GI symptoms	4 yr of follow-up No recurrence of bleeding At 2 yr of follow-up presented with vomiting and abdominal pain. Parents did not permit EGD, and treatment for EG was reintroduced empirically with ketotifen and her previous ED for 3 mos. She has been symptom-free for 2 yr after the second treatment.	3 yrs of follow-up No recurrence of bleeding No GI symptoms Persistent AR, for which he receives nasal steroids when needed.

AD = atopic dermatitis; AR = allergic rhinitis; CRP = C-reactive protein; ED = elimination diet; EG = eosinophilic gastritis; EGD = eosinophilic gastrointestinal disease; EoE = eosinophilic esophagitis; Hb = hemoglobin; HPI = history of presenting illness; GI = gastrointestinal; OVB = oral viscous budesonide; PE = peripheral eosinophilia; PEC, peak eosinophil counts; PEG = polyethylene glycol; SPT, skin prick testing; PPI = proton pump inhibitor; WBC = white blood cell.

### Cases With EG/EGE

Two patients (cases 4 and 5) presented to the emergency department with hematemesis. Case 4 had a history of constipation, and case 5 was experiencing recurrent AP and feeding refusal for 6 months before the presentation. Both had a history of atopic dermatitis, which resolved at 4 years of age. One had current allergic rhinitis. Laboratory abnormalities were significant for anemia, leukocytosis, elevated CRP, and elevated serum IgE levels in both patients. Upper GI endoscopy revealed mucosal edema and severe hyperemia of the corpus (2), a large sheet erosion in antrum (1), and a gastric antral ulcer (1). Biopsies taken from the gastric mucosa were consistent with EG and showed cryptitis, inflammation, and gastric peak eosinophil counts of 98 and 126 eosinophils/hpf.

### Therapy

All patients were initially treated with IV proton pump inhibitor (PPI) and IV methylprednisolone at a dose of 1 mg/kg/d. Further treatment included oral PPI, oral viscous budesonide, and elimination diet (ED) in EoE and crushed budesonide and ED in EG/EGE. The EDs in EoE involved the empirical restriction of milk and wheat, as well as of the foods that were implicated by parent reports and skin prick testing. Polysensitization to food allergens were found in all 3 patients diagnosed with EoE empirical milk, wheat, and egg restrictions were used in EG/EGE patients.

### Short- and Long-term Follow-up

Duration of follow-up ranged between 2 and 6 years. Duration of treatment ranged between 2 and 12 months. Rebleeding was not observed. Symptomatic and endoscopic reevaluation was undertaken in all patients at 6 months of follow-up. Four patients exhibited clinical, endoscopic, and histologic remission at reevaluation, with complete resolution of eosinophilic inflammation. However, case 2 had persistent EoE with a peak eosinophil count decreased from his pretreatment baseline but still higher than 15 eosinophils/hpf. Oral viscous budesonide was continued for another 6 months, and histologic remission was achieved at 12 months.

### Review of the Literature

A PubMed search with the keywords “hematemesis,” “bleeding,” “melena,” “eosinophilic esophagitis,” “eosinophilic gastritis,” “eosinophilic gastroenteritis,” and “eosinophilic colitis” was conducted and revealed 16 publications dating between 2007 and 2020 (3–5, 8–20). A total of 26 patients, between 1 day and 75 years of age, who presented with UGIB or LGIB and received an EGID diagnosis, were identified. Among these, there were 2 patients with EoE; 19 patients with EG/EGE; 3 patients with EC; 1 patient with EoE and EGE; and 1 patient with EoE, EGE, and EC. Hemodynamic instability at admission was observed in only 4 of the cases. Elevated IgE levels and PE were reported in 3/6 and 5/11 of the cases, respectively. Allergen sensitization was found in 7/8 patients. Endoscopic or radiologic treatment was carried out in 3 cases. Medical treatment consisted of PPI, antihistamines, systemic glucocorticoids, and ED. The prognosis was favorable in all of the case reports with no recurrence of bleeding, and histologic resolution in those who underwent control endoscopy (6/7). Studies included in the literature review are summarized in Table 2.

**TABLE 2.** Review of the literature on EGID-associated gastrointestinal bleeding

Year	Author	Age	Gender	Dx	Presentation	HD instability	Anemia	Total IgE	PE	Atopy	Source of bleeding	Endoscopic/IR treatment	Medical treatment	Prognosis
**2020**	Priyadarshni ^(6)^	28 years	M	EGE	Hematemesis, melena	Yes	Yes	N/A	N/A	Peanut allergy	Duodenal ulcer	Embolization of bleeding artery	PPI, prednisone	N/A
**2019**	Daidone ^(4)^	4 months	F	EG	Blood-stained vomiting followed by frank hematemesis	No	No	N/A	No	N/A	Gastric ulcer	None	PPI, amino acid-based formula	No recurrence, histologic resolution at 3 mo. CMPA reintroduced at 14 months without recurrence.
**2018**	Soriano-Ramos ^(9)^	4 months	M	EGE	Blood streaked vomiting	No	No	N/A	No	CMP sensitization	Gastric antral erosion	None	Elemental formula	No recurrence, no diet restrictions currently.
**2018**	Soriano-Ramos ^(9)^	2 months	F	EG	Blood streaked vomiting	Tachycardia	Yes	N/A	No	CMP sensitization	Diffusely erythematous gastric mucosa	None	Elemental formula	No recurrence, still on diet restriction.
**2018**	Gonzalez-Canalizo ^(11)^	15 years	F	EG	Hematemesis	Yes	Yes	N/A	No	Nut sensitization	Gastric ulcer	Adrenaline, ethoxysclerol, clips	PPI, ranitidine, ebastine, nut restricted diet	No recurrence, histologic resolution at 18 months.
**2017**	Debuf ^(10)^	1 day	M	EC	Hematochezia	No	Yes	No	Yes	N/A	Severe inflammation from anal margin up to 7 cm	None	Amino acid formula	No recurrence, not on any restriction
**2017**	Shetty ^(5)^	10 weeks	F	EG	Projectile hematemesis	No	Yes	N/A	N/A	N/A	Gastric antral erosion	None	PPI, elemental formula	No symptomatic recurrence on elemental formula.
**2017**	Chen ^(12)^	54 years	M	EG	Periumbilical pain and melena	No	No	Yes	No	House dust mite sensitization	Verrucous gastritis, but gastric and duodenal ulcerations found following initial biopsy	None	Oral methylprednisolone, montelukast	Histologic resolution at f/o
**2017**	Aydin ^(13)^	16 years	F	EoE	Hematemesis	Tachycardia	Yes	N/A	N/A	N/A	Erythematous esophagus, gastric and duodenal ulcers (but no gastric or duodenal eosinophilia; only esophageal eosinophilia)	None	PPI, antihistamine, egg and milk restriction diet	Melena following discontinuation of antihistamines. Reinstitution of antihistamines + diet for 1 year. No recurrence and histologic resolution at 6 months. Still on restriction diet.
**2015**	Yamazaki ^(14)^	Early teens	M	EoE, EGE	Hematemesis, melena	No	Yes	Yes	No	Hx of asthma; peanut, soybean, wheat, egg white sensitization	Duodenal ulcer	Clipping of visible vessel	PPI, prednisolone	Symptomatic and endoscopic resolution with histologic persistence. Still on PPI.
**2015**	Choi ^(15)^	75 years	F	EGE	Hematemesis, hematochezia	No	No	N/A	N/A	Positive patch test to Rhus	Friable duodenal mucosa with touch bleeding	None	Rhus avoidance	Improving endoscopic findings at 1 month follow-up.
**2014**	Raithel ^(16)^	22 years	M	EGE	Melena	No	Yes	Yes	Yes	Hx of atopic dermatitis, allergic rhinitis; SPT and sIge negative for food and aeroallergens but segmental gut lavage revealed elevated food-specific IgE.	Multiple gastric, duodenal, and jejunal ulcers	None	Restriction diet based on sensitizations found in gastric lavage, hypoallergenic formula, antihistamines, H2B, pancreatic enzymes	Symptomatic and endoscopic resolution with histologic persistence on diet restriction and H2B.
**2013**	Alabsi ^(17)^	1 day	M	EoE, EGE, EC	Frankly bloody stools	No	Yes	N/A	Yes	N/A	Erythema in stomach and duodenum, erythema and ulcers in rectosigmoid.	None	Amino acid formula and breastfeeding with MDR of CMPA. No recurrence.	
**2007**	Ohtsuka ^(18)^	1 day	M	EC	Hematochezia	No	No	No	Yes	N/A	Bloody, erythematous colonic mucosa around rectosigmoid	None	Elemental formula	No recurrence, not on any restriction
**2007**	Ohtsuka ^(18)^	1 day	F	EC	Hematochezia	No	No	No	Yes	N/A	Massive oozing from rectosigmoid colon	None	NPO	No recurrence, not on any restriction
**2018**	Hommeida (series of children with EoE) ^(19)^			EoE	1 out of 73 children with EoE presented with hematemesis.									
**2015**	Choi (series of infants and children with EGE) ^(20)^			EGE	7/8 infants diagnosed with EGE presented with hematemesis, and 2/8 presented with melena. No patient in the child group presented with hematemesis or melena.									All infants responded to CMP restriction (amino acid, elemental, or MDR)
**2014**	Ko (series of children with EG) ^(21)^			EG	3/13 children with EG presented with anemia or melena.									

CMPA = cow’s milk protein allergy; CMP = cow’s milk protein; Dx = diagnosis; EC = eosinophilic colitis; EGE = eosinophilic gastroenteritis; EG = eosinophilic gastritis; EoE = eosinophilic esophagitis; IR treatment = interventional radiologic treatment; HD instability = hemodynamic instability; H2B = histamine 2 receptor blocker; MDR = maternal diet restriction; PE = peripheral eosinophilia; PPI = proton pump inhibitor; SPT = skin prick testing.

## DISCUSSION

GIB associated emergency department visits have been estimated to annually affect 93.9/100,000 children in the United States (21). Common etiologies for GIB in the pediatric population vary depending on the age group. Although rare, EGIDs have been reported in association with GIB, especially in the context of nonesophageal EGID. Here, we describe the clinical, laboratory, endoscopic, and histologic findings that we observed in 5 patients with EGID-associated GIB, an uncommon yet treatable cause of GIB in the pediatric population. Furthermore, 3 patients in our series had GIB secondary to EoE, which has been reported seldom previously.

Patients with EGID present with chronic, nonspecific symptoms that are related to the dysfunction of the part of the GI tract involved. While feeding intolerance, vomiting, food impaction, and dysphagia have been associated with EoE; abdominal pain, nausea, vomiting, weight loss, and diarrhea have been described in the setting of EG/EGE (22). Hematemesis and melena have been described as a symptom of EoE and EG/EGE in 3 different case series, occurring in 1.4% of children with EoE, 23% of children with EG, and 87.5% of infants with EGE (18–20). Furthermore, several case reports have reported hematemesis, melena, and hematochezia in the setting of EGID (3, 5, 8). Given the high variability of symptoms, high rates of GIB observed in 2 case series and the steadily increasing incidence of EGID, EGID should be considered in the differential diagnosis of GIB, especially in patients with suggestive characteristics.

The pathogenesis underlying EoE and EG/EGE is not fully understood but has been described to involve a specific Th2-mediated and IL-5 driven eosinophilic inflammation in response to antigenic stimuli (2). A potential allergen-mediated pathogenesis is reflected by the high rates of coexisting allergic disease, PE, elevated serum IgE, and allergen sensitization observed in EoE and EG/EGE patients (7). Correspondingly, all 5 of our patients had a previous or current history of an allergic condition, PE, and elevated serum IgE levels. An analysis of previous case reports also revealed high rates of PE (5/11) and serum IgE elevation (3/6). Therefore, these parameters may guide the physicians toward EGID in a patient presenting with GIB. Nevertheless, it should be noted that the absence of these laboratory findings are not sufficient to rule out EGID as the rate of PE and IgE elevations are reported to be 50% and 60% in EoE, versus 80% and 68% in EG/EGE, respectively (23).

The diagnosis of EGID relies on clinicopathological correlation and requires endoscopic biopsy. Endoscopic findings in EGIDs include esophageal rings, furrows, and white exudates in EoE, and gastric pseudopolyps, erythema, erosions, and ulcers in EG/EGE (22). However, gross endoscopic findings may be normal even in the presence of histologic eosinophilic inflammation. Therefore, current guidelines recommend taking at least 5 esophageal biopsies in suspected EoE, and at least 4–5 segmental biopsies in suspected EG/EGE, especially in those with nonspecific gastrointestinal symptoms and peripheral eosinophilia (24, 25). While this principle should guide the diagnostic endoscopies undertaken to diagnose suspected EGID, 2 case reports have described the development of significant gastric bleeding and hematoma following upper endoscopy with biopsy in 2 patients who presented with GIB secondary to EG/EGE (3, 11). Although in cold mucosal biopsies, the risk of clinically significant bleeding is estimated to be less than 0.5% (26), patients with EGID may exhibit increased mucosal fragility promoted by the release of eosinophilic cytotoxic granular proteins such as major basic protein, eosinophil cationic protein, eosinophil peroxidase, and eosinophil derived neurotoxin (27). Care must be taken to balance these 2 caveats when managing a patient with bleeding secondary to suspected EGID.

Interestingly, we had 3 EoE and 2 EG/EGE patients presenting with hematemesis, whereas the majority of the patients reported in the reviewed case reports had EG/EGE associated UGIB. Only 2 patients, out of the 26 cases reported, had isolated EoE as the cause of their UGIB (12, 18). In general, hematemesis and melena have been described as an exceedingly rare manifestation of nonerosive, erosive, and infectious esophagitis except for in the setting of coagulation abnormalities (28, 29). Although the rate of EoE-associated GI bleeding observed in this study is higher compared with the case reports, small number of cases in this series limits the generalizability of this finding. Nonetheless, EoE should still be included in the differential diagnosis of hematemesis, especially in patients with a history of feeding difficulty, dysphagia, and food impaction.

The treatment of EGIDs shares the common goals of inducing and maintaining remission. The treatment approach in EGID involves ED and pharmacologic therapy with PPIs and steroids in EoE, and PPIs, steroids, antihistamines, and mast cell stabilizers in EG/EGE with variable outcomes (7, 22). We acutely treated all of our EGID patients with IV PPIs and methylprednisolone to quickly induce remission. This therapy was followed by a combination therapy with oral PPIs, steroids, and ED. Our experience was similar to those observed in the pediatric EGID series by Ko et al with resolution of symptoms and histologic abnormalities at the end of the treatment period in most patients (20). Previous case reports of patients with EGID-associated GI bleeding have also demonstrated high rates of resolution and nonrecurrence of GIB with different types of ED. Although these findings are subject to publication bias, they are further supported by the findings of our case series, to reliably suggest that prognosis and treatment response are favorable in patients with EGID-associated GIB despite their more severe presentation. However, although the bleeding did not recur in any of our patients and the response to initial therapy was favorable in all cases, 3 patients experienced recurrent GI dysfunction 2–3 years following their initial presentation and received another course of the aforementioned treatment. Therefore, disease recurrence might be a potential complication that can be expected during the long-term follow-up of these patients. Fortunately, none of these patients showed signs of recurrence after the second course of treatment.

The strengths of this study include the presentation of long-term follow-up data and the objective demonstration of treatment efficacy with follow-up endoscopy and biopsy at 6 months. The limitations can be summarized as small number of cases due to the rarity of EGID-associated bleeding and the lack of a control group.

## CONCLUSION

EGID can present at any age with nonspecific signs and symptoms, which include upper and lower GI bleeding. GIB can be observed even in the absence of typical symptoms of EGID, such as chronic dysphagia. Accompanying allergic diagnoses, PE, and elevated IgE levels can be common. Definitive diagnosis is made with endoscopy and biopsy, which needs to be sampled from multiple sites due to the localized nature of disease involvement, and care must be taken in doing so since previous reports have described rebleeding associated with biopsy in EGID patients. Both our experience and previous case reports have described that in those presenting with GI bleeding due to EGID, response to combination medical and dietary therapy is favorable.
